# Blockade of sodium‑calcium exchanger via ORM-10962 attenuates cardiac alternans

**DOI:** 10.1016/j.yjmcc.2020.12.015

**Published:** 2021-04

**Authors:** Jozefina Szlovák, Jakub Tomek, Xin Zhou, Noémi Tóth, Roland Veress, Balázs Horváth, Norbert Szentandrássy, Jouko Levijoki, Julius Gy. Papp, Neil Herring, András Varró, David A. Eisner, Blanca Rodriguez, Norbert Nagy

**Affiliations:** a Department of Pharmacology and Pharmacotherapy, Faculty of Medicine, University of Szeged, Hungary; b Department of Physiology, Anatomy, and Genetics, University of Oxford, United Kingdom; c Department of Computer Science, University of Oxford, United Kingdom; d Department of Physiology, Faculty of Medicine, University of Debrecen, Hungary; e Faculty of Pharmacy, University of Debrecen, Hungary; f Orion Pharma, Espoo, Finland; g MTA-SZTE Research Group of Cardiovascular Pharmacology, Hungarian Academy of Sciences, Szeged, Hungary; h Unit of Cardiac Physiology, Manchester Academic Health Science Centre, University of Manchester, Core Technology Facility, Manchester, UK

**Keywords:** Alternans, Sodium‑calcium exchanger, Sodium‑calcium exchanger inhibition, Canine myocytes, Cardiac simulation

## Abstract

Repolarization alternans, a periodic oscillation of long-short action potential duration, is an important source of arrhythmogenic substrate, although the mechanisms driving it are insufficiently understood. Despite its relevance as an arrhythmia precursor, there are no successful therapies able to target it specifically. We hypothesized that blockade of the sodium‑calcium exchanger (NCX) could inhibit alternans. The effects of the selective NCX blocker ORM-10962 were evaluated on action potentials measured with microelectrodes from canine papillary muscle preparations, and calcium transients measured using Fluo4-AM from isolated ventricular myocytes paced to evoke alternans. Computer simulations were used to obtain insight into the drug's mechanisms of action. ORM-10962 attenuated cardiac alternans, both in action potential duration and calcium transient amplitude. Three morphological types of alternans were observed, with differential response to ORM-10962 with regards to APD alternans attenuation. Analysis of APD restitution indicates that calcium oscillations underlie alternans formation. Furthermore, ORM-10962 did not markedly alter APD restitution, but increased post-repolarization refractoriness, which may be mediated by indirectly reduced L-type calcium current. Computer simulations reproduced alternans attenuation via ORM-10962, suggesting that it is acts by reducing sarcoplasmic reticulum release refractoriness. This results from the ORM-10962-induced sodium‑calcium exchanger block accompanied by an indirect reduction in L-type calcium current. Using a computer model of a heart failure cell, we furthermore demonstrate that the anti-alternans effect holds also for this disease, in which the risk of alternans is elevated. Targeting NCX may therefore be a useful anti-arrhythmic strategy to specifically prevent calcium driven alternans.

## Highlights

• Cardiac alternans is a major proarrhythmic phenomenon with no specific treatment.
• We demonstrate that NCX blockade via ORM-10962 attenuates alternans.
• Three distinct morphological types of APD alternans observed.
• Human cardiac simulations provide a mechanism of alternans attenuation by ORM-10962.
• This is mainly due to reduced refractoriness of calcium cycling in the SR.

## Graphical abstract

**Figure:**
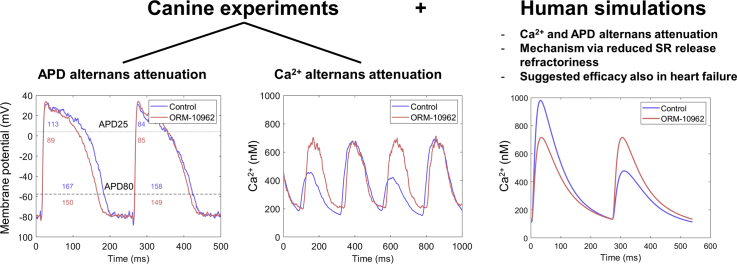

## 1 Introduction

The cardiac sodium‑calcium exchanger (NCX) exchanges a single calcium ion for three sodium and is therefore electrogenic [1]. It links cardiac electrophysiology with cellular calcium handling as NCX is the primary path of calcium efflux from the cell [2]. The exchange may proceed in either direction, depending on ionic concentrations and membrane potential: *forward* mode (calcium efflux) or *reverse* mode (calcium influx). Given its central role in cellular function, it is not surprising that NCX is implicated in multiple arrhythmogenic phenomena, such as early afterdepolarizations [3], delayed afterdepolarizations [4], and alternans [5]. Experimental research of NCX was until recently complicated by a lack of specific NCX blockers [6]. However, the NCX blocker ORM-10962 has been shown to be a useful tool for the study of NCX, given its selectivity and lack of strong preference to either mode of NCX [6]. While this drug does not block the L-type calcium current (I_CaL_), it was nevertheless observed to reduce it indirectly, likely via calcium-driven inactivation [7]. ORM-10962 has been applied to the study of afterdepolarizations [6], but not cardiac alternans, which is the focus of this study.

Action potential duration (APD) alternans is the oscillation of long and short APD at rapid heart rates, and has been shown to precede the formation of arrhythmia in the heart [8,9]. Multiple studies suggest that the mechanisms of arrhythmia induction following alternans are linked to increased dispersion of repolarization [[10], [11], [12]]. The vulnerability of hearts to alternans considerably increases in many heart diseases, such as heart failure [13], hypertrophic cardiomyopathy [14], or myocardial infarction [12,15,16]. APD alternans typically occurs concurrently with oscillation of calcium transient (CaT) amplitude [17,18], presenting the question which of these two is the main driver. It is possible that steep restitution of APD drives alternans [19]. However, further studies suggested that CaT oscillations are the primary driver [20], subsequently translated into APD alternans by NCX and other calcium-sensitive currents [21].

Given the possible involvement of NCX in alternans, ORM-10962 may be a promising antiarrhythmic strategy, particularly in cardiac diseases with elevated risk of alternans. However, the precise effect of NCX blockade on alternans is not necessarily straightforward. If we assumed a constant level of CaT alternans, NCX blockade would clearly reduce APD alternans. However, the assumption of constant CaT alternans following NCX blockade may not hold. On the one hand, it has been suggested that CaT alternans occurs if there is a steep dependence of calcium efflux from the cell on the SR calcium content [22]. Inhibition of NCX will make this dependence less steep and might therefore decrease alternans. On the other hand, CaT alternans is mostly thought to arise from increased sarcoplasmic reticulum release and CaT amplitude [11,23]. Therefore, the effect of NCX block, reducing efflux and locking more calcium in the cell, could be an increase in SR loading and thus release, increasing alternans. This could subsequently negate the reduction in APD alternans, or even extend the presence of alternans to slower pacing rates, which would be proarrhythmic. The aim of this study was therefore to characterize the effect of NCX blockade on both APD and CaT alternans and provide insight into the mechanisms involved.

## 2 Materials and methods

### 2.1 Experimental methods

#### 2.1.1 Ethical statement

All experiments were carried out in compliance with the Guide for the Care and Use of Laboratory Animals (USA NIH publication No 85–23, revised 1996) and were approved by the Csongrád County Governmental Office for Food Safety and Animal Health, Hungary (approval No.: XIII/1211/2012). The ARRIVE guidelines were adhered to during the study, (NC3Rs Reporting Guidelines Working Group, 2010).

#### 2.1.2 Standard microelectrode technique

Beagle dogs of either sex weighing 8 to 16 kg obtained from a licensed supplier were used for the study. Following sedation of the dogs with xylazine (1 mg/kg, *i.v.)* and thiopental (30 mg/kg *i.v.*), each heart was rapidly removed through a right lateral thoracotomy and immediately rinsed in oxygenated Locke's solution. Action potentials were recorded at 37 °C from the surface of dog right ventricular papillary muscles using conventional microelectrode technique. A total of *n* = 13 preparations were recorded from *n* = 9 hearts, and clustering analysis was carried out to ascertain that pseudoreplication did not occur when more than one preparation per heart was measured (Appendix F). The preparations were mounted in a custom made plexiglass chamber, allowing continuous superfusion with O_2_-CO_2_ saturated Locke's solution (containing in mM: NaCl 120, KCl 4, CaCl_2_ 1.0, MgCl_2_ 1, NaHCO_3_ 22, and glucose 11). The pH of this solution was set between 7.35 and 7.4 when saturated with a mixture of 95% O_2_ and 5% CO_2_ at 37 °C. The tissue samples were stimulated with constant current pulses of 1 ms duration at a rate of 1 Hz through a pair of bipolar platinum electrodes using an electrostimulator (Hugo Sachs Elektronik, model 215/II). The stimulus electrode was placed at the proximal site of the papillary muscle providing impulse propagation towards the tendons. Sharp microelectrodes with tip resistance of 10–20 MΩ, when filled with 3 M KCl, were connected to an amplifier (Biologic Amplifier, model VF 102). Voltage output from the amplifier was sampled using an AD converter (NI 6025, Unisip Ltd). Alternans was evoked by application of 20 beats at cycle lengths of 250 ms to 160 ms. Efforts were made to maintain the same impalement throughout the whole experiment. When the impalement was dislodged, an adjustment was attempted. The measurements were only continued if the action potential characteristics of the re-established impalement deviated less than 5% from the previous one. APD alternans were measured at a following pre-defined pacing cycle lengths: 250, 230, 210, 190 and 170 ms. After recording the control pacing protocol, 1 μM ORM-10962 was employed and alternans protocol was repeated.

#### 2.1.3 Cell isolation

Ventricular myocytes were enzymatically dissociated from dog hearts. The excised left ventricular segments were perfused through the left anterior descending coronary artery using a gravity flow Langendorff apparatus. The perfusate was a modified Tyrode solution (composition in mM: NaCl 144, NaH_2_PO_4_ 0.4, KCl 4.0, MgSO_4_ 0.53, glucose 5.5) supplemented with 1.2 mM CaCl_2_, 10 mM HEPES, 20 mM taurine, and 1.6 mM Na-pyruvic acid (pH = 7.2 with NaOH). After removal of the blood the perfusate was switched for 10 min to nominally Ca^2+^-free Tyrode. Dispersion of cells was achieved by application of 0.5 g/l collagenase (Sigma type I) for 40 min in the presence of 50 μM CaCl_2_. During the isolation procedure the solutions were gassed with 100% oxygen and the temperature was maintained at 35 °C. Finally, the tissue was minced and gently agitated. The cells, freshly released from the tissue, were stored at room temperature before use. At least 60% of the cells were rod-shaped and showed clear striation when the external Ca^2+^ was restored. One drop of cell suspension was placed in a transparent recording chamber mounted on the stage of an inverted microscope (Olympus IX71, Olympus, Tokyo, Japan), and individual myocytes were allowed to settle and adhere to the chamber bottom for at least 5–10 min before superfusion was initiated and maintained by gravity. Only rod-shaped cells with clear striations were used. HEPES-buffered Tyrode's solution served as the normal superfusate.

#### 2.1.4 S1S2 restitution

In order to determine the recovery kinetics of APD_80_ extra test action potentials were elicited by using single test pulses (S2) in a preparation constantly paced in a basic cycle length of 1000 ms. The S1-S2 interval was increased progressively from APD_80_ − 50 ms to APD_80_ + 500 ms. The effective refractory period was defined as the longest S2 coupling interval that did not lead to an action potential developing in the tissue sample. In addition, postrepolarization refractoriness (PRR) was inferred from the S1S2 restitution protocol. PRR was defined as the shortest diastolic interval between the S1 and S2 stimuli that led to a propagation of an action potential (this corresponds to ERP - APD(S1), where APD(S1) is the APD80 of the S1 stimulus and ERP is the effective refractory period).

#### 2.1.5 Calcium transient measurements

CaTs were recorded using a Ca^2+^-sensitive fluorescent dye, Fluo-4 AM simultaneously with ionic current measurements. Cardiomyocytes isolated from the left ventricle were loaded with 5 μM Fluo 4-AM for 15 min at room temperature in the dark. Loaded cells were mounted in a low volume imaging chamber (RC47FSLP, Warner Instruments) and field stimulated with a pair of platinum electrode. Frequency pattern was applied by using Evokewave v1.49 software. Fluorescence measurements were performed on the stage of an Olympus IX 71 inverted fluorescence microscope. The dye was excited at 480 nm and the emitted fluorescence is detected between 515 and 550 nm. Optical signals were sampled at 1 kHz and recorded by a photon counting photomultiplier module (Hamamatsu, model H7828). Data acquisition and analysis were performed using Axon Digidata 1550B System. All CaTs were measured at 37C°. In order to calibrate the records, alterations in Ca^2+^_i_ were calibrated after disruption of the cells by a patch pipette, by using the following equation for Fluo-4:

$${{\mathrm{Ca}}^{2+}}_{i}=\mathrm{Kd}*\left(F-F_{\min}\right)/\left(F_{\max}-F\right)$$

### 2.2 Signal analysis

APD alternans was extracted based on four consecutive action potentials as the difference between average APDs of even and odd AP. The electrophysiological measurements were at most 1 s long, yielding four action potentials at the longest cycle length of 250 ms. A custom-written Matlab script was used for this purpose. The four beats were smoothed via zero-phase digital filtering (@filtfilt in Matlab) with diameter of 10 ms. The baseline of such signal was taken as the 2.5-percentile of the signal, and the peak as the 99.5-percentile. Based on this, the thresholds (membrane potential levels at which APD is measured) for APD25 (25% repolarization, i.e., mainly plateau duration) and APD80 (80% repolarization) were computed. Each threshold was applied to the whole 4-beat recording, rather than being estimated from each AP separately. This is to avoid “threshold alternans” where alternation of AP peak potential induces alternation of the thresholds, which taints the estimation of the degree of alternans. In the majority of recordings of APD alternans, the basic cycle lengths used were 170, 190, 210, 230, and 250 ms. In several preparations, slightly different basic cycle lengths were used – in such a case, the alternans at the above-mentioned basic cycle lengths was obtained interpolation (e.g., *alternans* at 170 ms basic cycle length would be obtained as the average of alternans at 160 and 180 ms if these two were available). When diastolic intervals were measured, they were taken as the periods between segments corresponding to APD80 of action potentials. The first and last such diastolic intervals were discarded as they are typically incomplete.

Calcium alternans in the experiments was based on six consecutive CaTs, where the average CaT amplitude was computed for even and odd beats. The signal was separated into CaTs using the ‘comb’ algorithm for fixed-rate pacing [24]. The amplitude of a single CaT was estimated as the difference between the peak and the minimum directly preceding the given CaT (i.e., it is the CaT upstroke amplitude). CaT alternans is expressed as the ratio of the amplitudes of the larger to the smaller CaT.

Three subtypes of APD alternans were defined based on the phase of alternans at the levels of 25% and 80% repolarization:

• Recordings of the '++' alternans type manifest concurrent prolongation (or concurrent shortening) by at least 3 ms both at the 25% and 80% repolarization level. I.e., oscillations of APD at the 25% and 80% levels are in phase.
• Recordings of the '+−' alternans manifest concurrent prolongation by at least 3 ms at the 25% repolarization level and shortening by at least 3 ms at the 80% repolarization level (or vice versa). I.e., oscillations of APD at the 25% and 80% levels are in the opposing phase.
• Recordings of the '+0' are recordings manifesting shortening or prolongation by at least 3 ms at the 25% repolarization level, while the prolongation or shortening at the 80% level is less than 3 ms. I.e., there is clear APD25 alternans, but not APD80 alternans.

When analysing experimental S1S2 restitution protocol, the APD was measured at 80% level of repolarization using Evokewave v1.49 software (Unisip Ltd).

### 2.3 Computer simulations

ToR-ORd, a recent robustly validated human ventricular myocyte model, was used throughout this study [25], being selected for its improved representation of L-type calcium current and sodium and calcium balance compared to its predecessors. This model is capable of reproducing calcium-driven alternans stemming from refractoriness of Ca^2+^ cycling in the sarcoplasmic reticulum; the mechanism is described in detail in [23]. It is therefore more suitable for this study than other human ventricular models, such as Grandi et al. [26], which does not manifest alternans, or Ten Tusscher et al. [27], which manifests alternans, but one that is driven by APD restitution (which is shown not to be the key cause of alternans in the experiments in this study). Simulations using a human model provide a bridge for the interpretation and translation of the findings obtained using animal experiments towards clinically-relevant outcomes. The effect of ORM-10962 on ionic dynamics was modelled as reducing NCX availability to 50% and reducing I_CaL_ availability to 65% as observed experimentally [7]. Additional experiments were carried out to verify that even at rapid pacing used in this study, the NCX block achieved with the given ORM-10962 concentration is close to 50% (Appendix G).

Originally, we also considered the most up-to-date canine model of the Rudy family [28]. However, when simulating the effect of ORM-10962, the canine model predicted a substantial APD prolongation not seen in our experimental data (see Fig. 3; mean APD at DI of 500 ms is 183 ± 31 ms for ORM-10962, and 198 ± 23 ms for control; *p* = 0.28) or that of others [6]. Subsequent analysis revealed that the APD prolongation in the canine model is linked to artefactual lack of I_Kr_ activation following AP plateau lowering (which follows from I_CaL_ reduction). This is a manifestation of a problem which warranted the replacement of the I_Kr_ model in development of ToR-ORd [25]. For this reason, and given that there are no known differences between canine and human calcium handling pertaining to the phenomena studied there, we believe that ToR-ORd is the best model choice.

The S1-S2 protocol was simulated by first prepacing the cell during 1000 beats at 1 Hz (S1), followed by a single S2 stimulus at varying S1-S2 intervals = 150, 152, 154, …350, and then 400, 500, 600, …, 1000. For this protocol, the I_Na_ conductance was reduced to 25% to more clearly demonstrate the role of I_CaL_ in cellular excitability (see Section 3.4 for more details). The reaction to S2 stimulus was classified as an action potential if the peak membrane potential exceeded 10 mV.

When evaluating ERP in single cell for the investigation of postrepolarization refractoriness, this was defined as the longest S2 coupling interval that did not trigger a S2 AP.

#### 2.3.1 Population of models construction

A population of ToR-Ord models was created by simulating 5000 models with perturbation of conductances of the following ionic currents: I_Na_, I_NaL_, I_CaL_, I_to_, I_NaK_, I_NaCa_, I_Kr_, I_Ks_, I_K1_. Each current in each model was sampled in the 50–200% range (corresponding to a multiplier of 0.5–2), using symmetric sampling where the probability of x is the same as 1/x. The formula used to obtain each multiplier is $e^{\left(U\left(01\right)-0.5\right)∙\frac{\log\left(2\right)}{0.5})}$, where U(0,1) is random number uniformly sampled between 0 and 1. It can be seen that e.g. for the random number being 0 and 1, the resulting values of multiplier are 0.5 and 2 respectively. This approach ensures that approximately half of multipliers is below 1 and a half is above 1 (as opposed to uniformly sampling between 0.5 and 2 straight away, which will assign only a third of generated values below 1, and two thirds above 1, overrepresenting current conductance increase on average).

A calibration to human experimental data was carried out using the following criteria:

• APD40, APD50, APD90, triangulation (APD90-APD40), peak upstroke velocity, and resting potential within experimental range, as in population of models construction in [29].
• Calcium transient duration is within standard deviation of the data in [30].
• The model manifests alternans at at least one of the tested basic cycle lengths (240, 250, …, 340 ms).

A total of 684 models fulfilled all the criteria, and these were subsequently used to compare alternans onset between the model and four simulated versions of NCX blockade (50% NCX availability + 50, 65, 70, and 80% I_CaL_ availability respectively).

#### 2.3.2 Heart failure simulations

Heart failure remodelling was introduced in the ToR-ORd model, mainly based on studies summarized and used in [31]. Similarly to their study, we increased the conductances of I_NaL_ by 80%, I_NaCa_ by 50%, I_Cab_ by 53%, and reduced the conductance of I_to_ by 60%, I_K1_ by 32%, and I_NaK_ by 30%. In addition, the time constant of I_NaL_ inactivation τ_hL_ was increased by 80%, CaMKII autophosporylation rate was increased by 50%, and J_leak_ (leak from NSR to main cytosolic compartment) by 30%. The rate of SR reuptake via SERCA pumps J_up_ was reduced by 30% and SR release was made 20% more sensitive to calcium in the junctional SR.

In addition, we added an explicit leak flux J_leak,JSR_ to the ryanodine receptor to represent increased ryanodine receptor leakiness present in heart failure [32]. The formulation is based on the J_leak,JSR_ in [28]:

$$e^{\frac{Ca_{\mathrm{JSR}}}{20}}∙\left(Ca_{\mathrm{JSR}}-Ca_{\mathrm{SS}}\right)$$

Ca_JSR_ is the calcium concentration in the junctional SR, and Ca_SS_ is the calcium concentration in the junctional subspace.

Furthermore, the time constant of SR release was increased six-fold to represent the phenotype of SR release present in heart failure, where calcium sparks have markedly longer time to peak and duration [33,34]. Its precise magnitude is however not known. This change is very important for the increased vulnerability of the simulated heart failure cell to alternans by mechanism described in [23].

Ultimately, the sensitivity of SR uptake to intracellular calcium concentration was reduced, as this was also observed in heart failure [35]. This was achieved by shifting the half-activation of the SERCA reuptake as follows (for nonphosphorylated and phosphorylated reuptake):

$$J_{\mathrm{up},\mathrm{NP}}=0.005425∙\frac{Ca_{i}}{Ca_{i}+0.00092+0003}$$

$$J_{\mathrm{up},P}=2.75∙0.005425∙\frac{Ca_{i}}{Ca_{i}+0.00075+0003}$$

The elements in bold were added to achieve the desensitization.

#### 2.3.3 S1S2 in simulated cardiac fibre

The 1D fibre tissue simulations were conducted using the open-source software CHASTE [36]. The homogeneous monodomain 1D endocardial fibres had a length of 4 cm consisting of 200 nodes, with a conductivity of 1.64mS/cm to achieve a diffusion coefficient of 1.171 cm2/s [37]. The 1D fibres were paced under an S1-S2 protocol: regular S1 stimulus was applied with a cycle length of 1000 ms for 20 beats, followed by a single S2 stimulus; the coupling intervals simulated were 150, 152, 154, …, 450, and then 500, 600, …, 1000. Restitution curves were measured at the centre of the fibre. As in single cell restitution, I_Na_ conductance was reduced to 25%.

When evaluating ERP in a fibre for the investigation of postrepolarization refractoriness, this was defined as the longest S2 coupling interval that did not trigger a S2 AP in the cell at position x = 2 cm.

### 2.4 Statistical methods

**Fig. 1:**
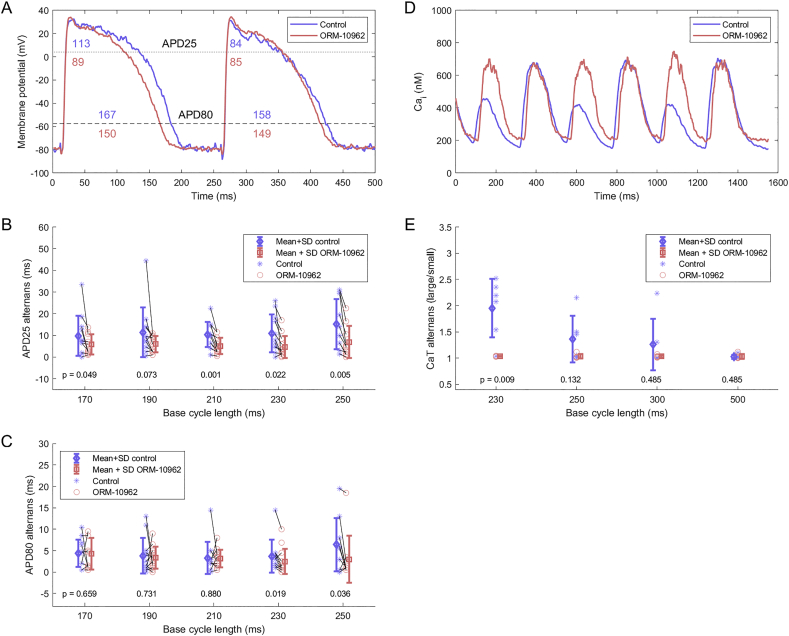
Alternans attenuation via ORM-10962. A) Example of APD alternans at two levels (APD25 and APD80) in control condition and following ORM-10962 exposure in muscle preparations. B) APD25 alternans at a range of pacing rates (*n* = 13). C) APD80 alternans at a range of pacing rates (n = 13). In B,C, paired *t*-test was used to compute the *p*-values. D) Example of reduction in CaT alternans in isolated cells via ORM-10962. E) Quantitative summary of CaT alternans (expressed as the ratio between the amplitude of the larger versus smaller CaT) without (*n* = 6) and with (n = 6) ORM-10962. Unpaired rank-sum test was used in E given different variance between control and ORM-10962.

Paired *t*-test was used for paired comparisons (Figs. 1B,C, 3B,C, 6A). Wilcoxon rank-sum test was used for unpaired comparisons (Fig. 1E), used over unpaired t-test given markedly different variance between the groups. The statistical significance level used was α = 0.05.

## 3 Results

### 3.1 NCX blockade via ORM-10962 inhibits alternans of APD and CaTA

APD alternans was measured in canine papillary muscle preparations at two repolarization levels (25% and 80%), both in control conditions and after the application of ORM-10962 (Fig. 1A). ORM-10962 significantly attenuated APD25 at most basic cycle lengths (Fig. 1B). Alternans of APD80 was significantly attenuated only at the slowest pacing (basic cycle lengths 230 and 250) (Fig. 1C). To complement the electrophysiological recordings of alternans, calcium imaging measurements were additionally taken in isolated cells. These recordings confirmed that calcium transient amplitude alternans was also potently and significantly attenuated by ORM-10962 at 230 ms bcl (Fig. 1D,E).

**Fig. 2:**
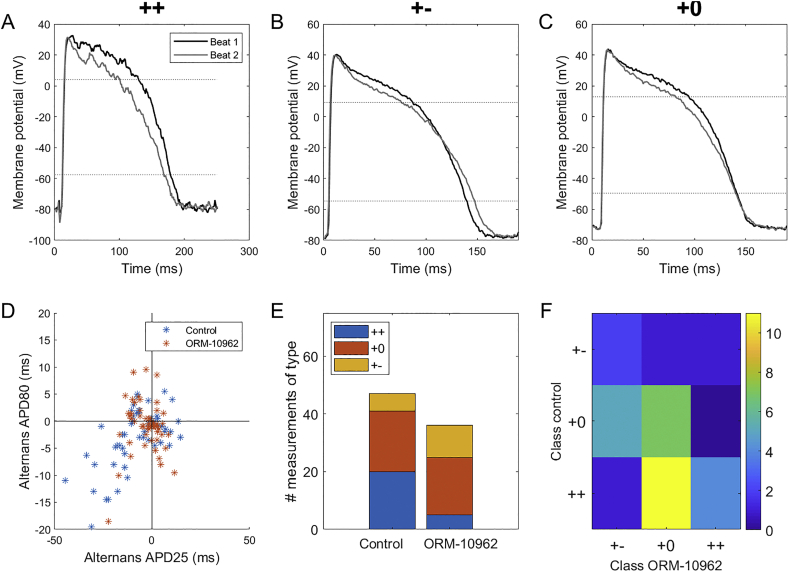
Distinct patterns of alternans at two repolarization levels. A-C) Examples of '++', '+−', and '+0' types of alternans respectively (i.e., shortening plateau corresponds to prolonged, shortened, or unchanged APD at 80% level of repolarization). The three recordings shown were obtained at bcls of 250, 190, and 190 ms respectively. D) Correlation of the difference in consecutive APDs at repolarization levels of 25% and 80%; all tested bcls were pooled together in this plot. E) Composition of the three alternans types in the control condition and following ORM-10962 treatment. The lower total number of entries following ORM-10962 stems from alternans attenuation by the compound: a recording has to manifest alternans of at least 3 ms at the APD25 level in order to be classified as one of the three types. F) A transition matrix encoding the change in alternans type upon ORM-10962 application. Each matrix element colour-codes the number of preparations manifesting a given type of alternans in the control condition (y-axis) that transitioned into a given type of alternans after ORM-10962 application (x-axis).

Investigating the relationship between APD25 and APD80 alternans, we observed three types of behaviour. In the '++' type, the APD is prolonged/shortened at both levels of repolarization, at the same time between subsequent action potentials (Fig. 2A). In type '+−', the APD is prolonged at one level, but shortened at the other one (Fig. 2B). Ultimately, in type '+0', there is visible alternans at the APD25 level, but none at APD80 (Fig. 2C). All three types were present in control and ORM-10962 conditions, with ORM-10962-treated cells showing fewer recordings of the '++' type and more of the '+−' type (Fig. 2D,E). The analysis of transitions of alternans types before and after ORM-10962 treatment shows that many '++' recordings changed into the '+0' type with ORM10962, while '+0' recordings either stayed in the same class or transitioned towards the '+− ' type (Fig. 2F). We can thus see a pattern where a given APD25 prolongation during alternans is associated with less APD80 prolongation or even with its shortening following ORM-10962 treatment.

We carried out additional exploration of the effect of ORM-10962 on the three different alternans types reported above (Appendix B). Consistent with near-universal alternans attenuation observed for APD25 (Fig. 1B), alternans was generally attenuated by ORM-10962 for all alternans types (Appendix A, Supplementary Fig. 1A). However, the situation is different for alternans of APD80, where there was a marked difference in response of preparations manifesting '++' and '+0' alternans before the drug treatment (the number of cases of '+−' alternans is too low to allow drawing conclusions). Whereas alternans was attenuated in the vast majority of cases of '++' alternans (18 attenuated, 1 unchanged, 1 promoted; mean response is attenuation by 4.9 ms), the situation is much less clear for the '+0' type (8 attenuated, 3 unchanged, 9 promoted; mean response is promotion by 1.47 ms) (Appendix A, Supplementary Fig. 1B). In addition, the '++' type was prevalent for the slower pacing rates (basic cycle length of 230 or 250), but not for the faster ones (Appendix A, Supplementary Fig. 1C), which helps to explain why APD80 alternans attenuation was observed only for the slower pacing rates in Fig. 1C. In terms of absolute magnitude, APD80 alternans was the greatest in the '++' type, while being by definition minimal in the '+0' type (Appendix A, Supplementary Fig. 1D).

### 3.2 Alternans attenuation via ORM-10962 is not due to restitution of APD

**Fig. 3:**
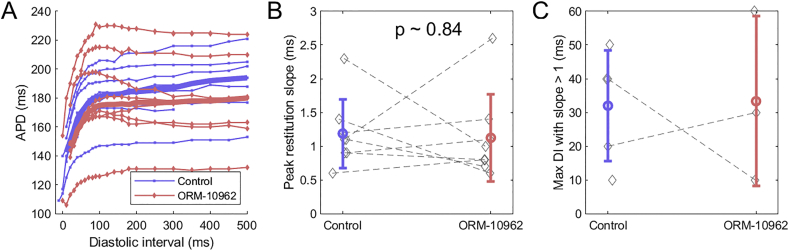
ORM-10962 and restitution properties. A) Restitution curves measured before and after application of ORM-10962 to canine tissue samples (*n* = 8). Thick lines give the median S2 APD (computed where at least 3 data points are available for the given diastolic interval). B) Paired measurements of maximum slope of the restitution curve before and after ORM-10962; the overlay gives mean ± SD. C) Longest diastolic interval before which the slope of restitution curve is greater than 1; the overlay gives mean ± SD.

We subsequently sought to determine the mechanism of alternans formation and its attenuation. We first explored the restitution-driven mechanism of APD alternans [19]. There the alternans arises from the slope of the restitution curve exceeding 1 for diastolic intervals (DI) between the alternating beats. We measured restitution curves using the S1-S2 protocol in canine ventricular muscle preparations (Fig. 3A). Before ORM-10962 application, 5 out of 8 hearts did contain an interval of the restitution curve with slope > 1 and 3 out of 8 contained such an interval after ORM-10962 application. The change in the peak slope after the application of ORM-10962 was not statistically significant (Fig. 3B, p = 0.84). The longest diastolic interval with slope > 1 was extracted in each recording (Fig. 3C), with the hypothesis that if diastolic intervals observed during alternans fall within the zone of restitution slope > 1, the restitution is a plausible explanation of APD alternans. The fact that in no recording the zone of slope > 1 extended beyond DI = 60 ms essentially excludes the restitution-based mechanism as the driver of alternans in our data, given that DI < 60 ms were rarely observed in alternans recordings. Only 4 out of 68 recordings of bcl ≥ 210 ms had the shorter DI below the observed maximum of 60 ms, and none had the shorter DI below 35 (median of points shown in Fig. 3C; both groups pooled). APD alternans was also present at 250 ms bcl pacing, where the shorter DIs are typically close to 100 ms, i.e., both short and long DI were outside the zone of steep restitution. Together, these observations indicate that alternans we observed is not driven by APD restitution, but potentially by calcium oscillations.

### 3.3 Computational simulations illuminate the role of altered balance of calcium handling in alternans attenuation

**Fig. 4:**
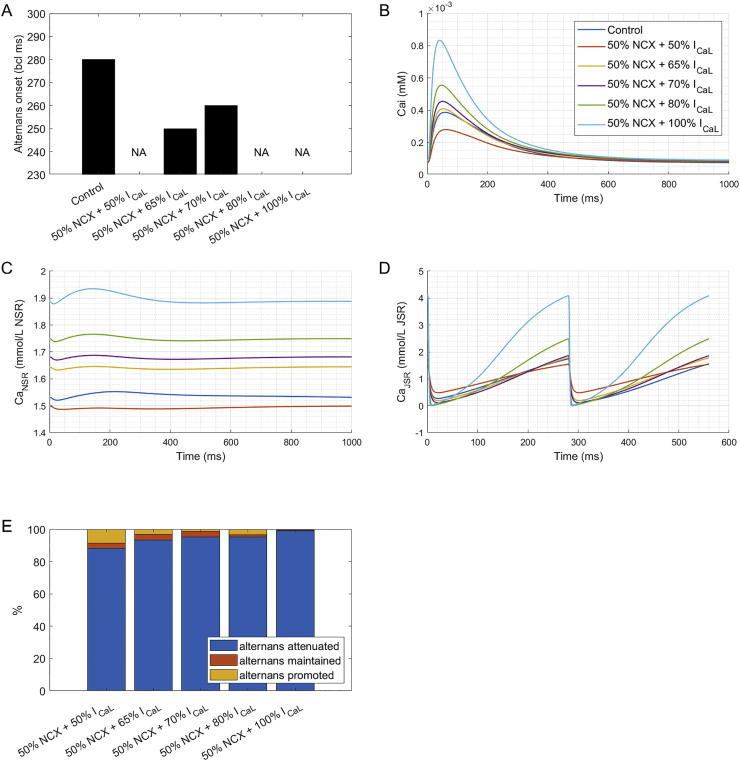
Simulations of alternans and ORM-10962. A) The longest basic cycle length at which was alternans detected in control condition and several combinations of NCX and I_CaL_ availability representing simulated ORM-10962 (NA = not available = no alternans). See Appendix C for a full frequency-alternans relationship at all the studied conditions. B) Calcium transient at 1000 ms bcl pacing. C) Calcium concentration in the network SR at 1000 ms bcl pacing. D) Calcium concentration in the junctional SR at 280 ms bcl pacing. E) A breakdown of the effect of five treatments simulating ORM-10962 (50% NCX availability and the five listed availabilities of I_CaL_) compared to the baseline model using a population of models approach. For each of 684 tested models in the population and for each treatment, a simulation was classified as ‘alternans attenuated’ if the treatment shifted alternans onset to shorter basic cycle length, ‘alternans promoted’ if alternans onset was shifted to longer basic cycle lengths, and ‘alternans maintained’ if alternans first appeared at the same basic cycle length. See Appendix D for a breakdown of how much was alternans promoted or attenuated in each condition.

Using the human ventricular myocyte ToR-ORd model [25], we then ran simulations of the action of ORM-10962 on calcium-driven alternans to gain further mechanistic understanding into the attenuation of calcium-driven alternans. The control ToR-ORd model manifests calcium-driven alternans for base cycle length of 280 ms and faster (Fig. 4A). The mechanism is linked to refractoriness of SR calcium cycling: following a large release at rapid pacing, the junctional SR is not replenished fully before the next beat, leading to CaT amplitude oscillations [23]. ORM-10962 was represented as a combined reduction of NCX and I_CaL_ (Methods 1.3). To gain a broader picture of the role of I_CaL_ reduction, we simulated five different levels of I_CaL_ availability (50%, 65%, 70%, 80%, and 100%) in addition to a 50% reduction of NCX. Interestingly, all studied combinations of NCX and reduced I_CaL_ availability led to alternans attenuation as assessed by moving alternans onset to faster frequencies, or abolishing alternans altogether (Fig. 4A); however, the mechanisms are distinct.

The case closest to experimental measurements is 50% NCX reduction and 65–70% I_CaL_ availability, which exerts a small or moderate increase in CaT amplitude compared to control ToR-ORd cell at 1 Hz pacing (Fig. 4B). This agrees with a previous study on ORM-10962 showing only a minor increase in calcium transient amplitude [6] and experimentally observed 65% I_CaL_ availability for 50% NCX availability after ORM-10962 treatment [7], Appendix G. The mechanism of alternans attenuation in such a setting is best illustrated on the case of 65% I_CaL_ availability: even though the CaT amplitude is almost identical to the control cell, the calcium loading of the network sarcoplasmic reticulum is increased at all tested pacing rates (example at 1 Hz in Fig. 4C). Consequently, the gradient between the network and junctional SR is increased, allowing faster refilling of the junctional SR, reducing SR refractoriness, inhibiting alternans [23].

A substantial increase in CaT amplitude compared to control cell is observed when I_CaL_ availability is 80% or 100% (Fig. 4B), and in these cases, no alternans is found (Fig. 4A). Inspection of the contents of junctional SR at base cycle length 280 (where the control model already shows alternans) reveals that the junctional SR is fully depleted in both beats in such conditions (Fig. 4D). The phenomenon of full SR depletion and consequent alternans abolishment in simulations was characterised previously [23], but its physiological relevance is debatable. Importantly, we note that in the cases of 65% and 70% I_CaL_ availability described above, the junctional SR is clearly not fully depleted at 280 ms bcl (Fig. 4D), verifying that the full depletion of junctional SR is not the mechanism of alternans attenuation in these cases.

When the availability of I_CaL_ is low (50%), alternans is also never present (Fig. 4A). Given the CaT amplitude is considerably diminished (Fig. 4B), the lack of alternans is clear: calcium alternans stems from a greater calcium release, and when the release is not sufficiently large, there is no alternans. However, no such reduction in CaT amplitude was observed upon ORM-10962 application [6], and so this explanation can be excluded.

In order to assess the sensitivity of the results to specific parameter values in the ToR-ORd model, we further investigated the impact of simulated ORM-10962 on alternans using the population of models approach [29,38]. A population of 684 simulated cells calibrated to human experimental data was used (see Methods for details), and each cell was simulated in normal conditions and with five levels of I_CaL_ availability accompanying a 50% NCX block as above. In all five conditions, the vast majority of models showed alternans attenuation compared to the control model (over 88%, Fig. 4E), supporting the relevance of the anti-alternans effect of ORM-10962. Furthermore, additional analyses revealed that the mechanism underlying a substantial part of the cases of alternans promotion (including all the cases of alternans promotion at 50% or 65% I_CaL_) has debatable relevance (Appendix B).

### 3.4 Validation of the anti-alternans effect of ORM-10962 in simulated heart failure

**Fig. 5:**
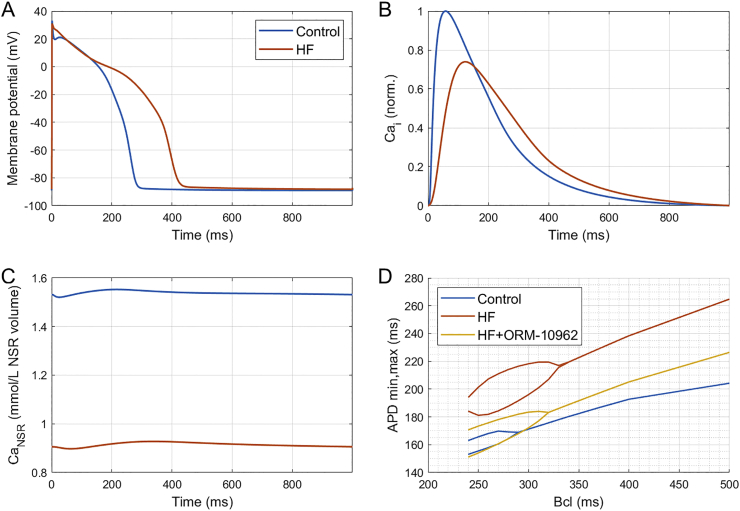
Simulations of heart failure remodelling and alternans. A) Action potential in control and heart failure (HF) condition at 1 Hz pacing, showing APD prolongation and loss of notch following the peak in HF. B) Calcium transient in control and HF, showing its prolongation, prolonged time to peak, and reduced amplitude. The calcium transient was normalised between 0 and 1 to facilitate comparison: first, the minimum was subtracted from both calcium transients; second, both calcium transients were divided by the maximum of the control trace, preserving the ratio of amplitude between control and HF. C) Calcium concentration in the network SR showing reduced SR loading in HF. D) Simulation of the effect of heart failure (HF) remodelling and HF + ORM-10962 treatment (50% NCX + 65% I_CaL_) on alternans development. For each model, the minimum and maximum APD90 achieved over two subsequent beats at the given basic cycle length is given, with bifurcations indicating alternans. Small-amplitude alternans in the HF condition developed already at bcl of 330 ms.

Even though alternans can usually arise even in healthy myocardium, its importance is even greater in cardiac diseases such as heart failure with reduced ejection fraction, where it manifests at slower pacing rates, often preceding the formation of ventricular fibrillation [8,13]. To assess the anti-alternans potential of ORM-10962 in heart failure, we incorporated a broad range of experimental data on heart failure remodelling in the baseline ToR-ORd model (see Methods). The resulting model replicates key hallmarks of human heart failure, such as APD prolongation and loss of spike-and-dome morphology (Fig. 5A) [39], reduced amplitude of calcium transient, along with the prolongation of its duration and time to peak (Fig. 5B) [40], and reduced calcium loading of the sarcoplasmic reticulum (Fig. 5C) [39].

Importantly, the onset of alternans in the control model (280 ms bcl) is shifted by heart failure remodelling to slower frequencies (330 ms bcl) (Fig. 5D), consistent with experimental observations [8,13]. The amplitude of repolarization alternans is also increased by the heart failure remodelling, leading to potentially greater dispersion of repolarization induced by alternans and thus a higher arrhythmic risk. Crucially, simulated ORM-10962 (using the data-driven version of 50% NCX and 65% I_CaL_ availability) attenuated alternans in the failing cell, both with regards to the alternans onset (310 versus 330 ms) and amplitude at most basic cycle lengths (Fig. 5D).

### 3.5 ORM-10962 extends post-repolarization refractoriness

**Fig. 6:**
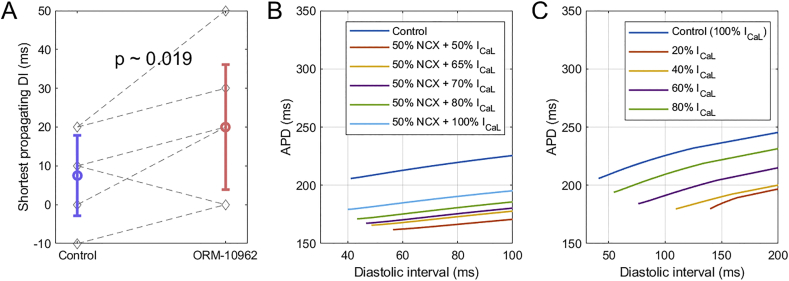
ORM-10962 extends PRR. A) Experimental data from papillary muscle preparations of the shortest DI which led to formation of an action potential in the S1-S2 protocol (*n* = 6). B) Simulations of S1-S2 protocol under NCX block and I_CaL_ reduction. The leftmost point on each curve determines the PRR in the given condition. C) Simulations of S1-S2 protocol where the S1 phase was carried out using control model without any perturbation of NCX and I_CaL_, with the I_CaL_ reduction being introduced only for the S2 beat.

Using the S1-S2 protocol, we observed that ORM-10962 extended the duration of post-repolarization refractoriness (PRR; see Methods 1.1.4 for the exact definition) (Fig. 6A). We hypothesized that this could be mediated by an indirect reduction of I_CaL_ via ORM-10962, given the current's involvement in cellular excitability [[41], [42], [43]]. The hypothesis was tested using the ToR-ORd model with an increased role of I_CaL_ in excitability (Methods 2.3).

The S1-S2 protocol was simulated using single cells with a 50% block of NCX and several possible values of reduced I_CaL_ availability. We observed that the lower the I_CaL_ availability, the longer the PRR (Fig. 6B); this result was also confirmed in a fibre simulation (Appendix E). To exclude the possibility that this is due to a chronic effect following from pre-pacing in different conditions (e.g. APD change or a change in ionic concentrations), an additional modified S1-S2 protocol was simulated. There, S1 pacing was carried out in a control ToR-ORd model, with I_CaL_ availability changed only for the S2 stimulus. Again, cells with reduced I_CaL_ manifested extended PRR (Fig. 6C). In both scenarios, this is due to the fact that for a short coupling interval, I_Na_ is reduced due to partial refractoriness, giving greater importance to I_CaL_ in triggering an action potential. When I_CaL_ is reduced, so is the cellular excitability. Together, these results demonstrate that the experimentally observed extension of PRR under ORM-10962 can stem from an indirect reduction of I_CaL_.

## 4 Discussion

The main findings of this combined experimental and computational work are: 1) ORM-10962 attenuates alternans of both action potential duration and calcium transient amplitude; however, the protective effect is not observed for APD80 alternans at very fast pacing rates. 2) Three distinct types of APD alternans are observed, revealing type-specific response to ORM-10962 with regards to APD80 alternans attenuation. 3) ORM-10962 does not markedly change APD restitution, and analysis of diastolic intervals suggests that calcium oscillations drive alternans in the experiments. 4) the suggested mechanism of calcium alternans inhibition by ORM-10962 is via reduced refractoriness of sarcoplasmic reticulum release. 5) ORM-10962 increases post-repolarization refractoriness, and this may be mediated by indirect L-type calcium current reduction following NCX blockade.

Cardiac alternans is an important arrhythmogenic phenomenon, which can be evoked in healthy hearts [44], but is particularly exacerbated in heart disease [[12], [13], [14]]. Alternans attenuation is consequently an important aim of antiarrhythmic medication, previously targeted mainly by SERCA pump overexpression [45]. Here, we demonstrate that the NCX blocker ORM-10962 has the potential to attenuate both alternans of APD and of calcium transient amplitude, with the alternans being calcium-driven. Importantly, our simulations suggest that ORM-10962 alternans attenuation could be effective also in the setting of heart failure remodelling.

In this study, we observed three types of APD alternans with regards to action potential morphology: '++' (APD25 prolongation during alternans occurs in the same beats as APD80 prolongation), '+−' (APD25 prolongation occurs in the same beats as APD80 shortening), and '+0' (APD25 alternans occurs in presence of negligible APD80 alternans). The fact that three distinct morphological patterns of alternans were observed in a single batch of experiments (in a single species and a single experimental protocol) suggests at relatively high and perhaps surprising intra-species phenotypic heterogeneity. ORM-10962 showed a tendency towards shifting alternans type to more negative type ('++' to '+0' and '+0' to '+−') compared to control. Response to ORM-10962 with regards to APD80 alternans differed by the type of alternans. While APD80 attenuation occurred for the vast majority of '++' alternans recordings, '+0' recordings have shown minimal attenuation or even alternans promotion (there was not enough data on '+−' type to draw any conclusions). Lack of alternans attenuation at APD80 for '+0' alternans is not surprising, given that the '+0' type is defined as type with little to no alternans at APD80. Consequently, a shift towards the '+−' type means that alternans at APD80 can be paradoxically promoted by ORM-10962. On the other hand, the negative-shifting effect of ORM-10962 on the alternans type means that the drug can be especially potent for '++' alternans, given that it can attenuate underlying calcium alternans, as well as shift the APD80 alternans type towards '+0', reducing dispersion of repolarization. The '++' type of alternans is possibly the most frequently reported type of alternans, observed in human [44], guinea pig [46,47], cat [48], or rabbit [17,49], supporting the relevance of alternans attenuation in this type. Finally, the fact that the '++' type manifested the greatest absolute amplitude of APD80 (with the amplitude being small or minimal for '+−' and '+0' types) means that clinical testing based on T-wave alternans is likely to detect precisely the patients who will benefit the most from hypothetical treatment based on NCX blockade. However, further research is needed to better characterize and understand the mechanisms (e.g. balance of ionic currents or regional differences) underlying the different types of alternans observed in this study.

The experimental results presented in this study suggest that ORM-10962 attenuates CaT alternans (as evidenced by measurements in isolated myocytes and by attenuation of APD25 alternans, which is very likely to be tightly associated with CaT alternans). This is an important result, given that analysis of diastolic intervals and restitution suggest that APD alternans in the presented experiments is not driven by restitution, but rather by alternans of CaT. The most likely explanation of calcium alternans attenuation suggested by computer simulations is the altered balance of two factors contributing to the amplitude of the calcium transient: the L-type calcium current and the SR calcium loading. While I_CaL_ is indirectly reduced by the NCX blockade [7], our simulations suggest that the SR loading is, in turn, increased. This prediction is supported by the fact that ORM-10962 reduces I_CaL_, but increases the calcium transient amplitude [6]. Therefore, there must be an additional contributor to the calcium transient amplitude which outweighs I_CaL_ reduction, and the increased SR loading is the most likely candidate. Such an effect is to be expected from an NCX blocker; the NCX and SERCA pumps in the SR are dominant mechanisms of removal of calcium from cytosol [2], and when NCX is inhibited, a greater relative role is given to SERCA pumps (Diaz et al., 2004), enhancing the reuptake into SR (provided the influx via I_CaL_ is not reduced excessively). Enhanced SR reuptake was then shown to reduce the refractoriness of calcium release from the SR [50]. Given that the refractoriness of release was shown to be a potential driver of alternans both experimentally and computationally [23,51], this could explain experimental observation of alternans inhibition following enhancement of SR reuptake [45,52].

Alternans attenuation via NCX inhibition may, in future, be an easier avenue towards alternans-aimed antiarrhythmics compared to previously considered SERCA overexpression. The NCX inhibition can be mediated by a channel-blocking drug such as ORM-10962, which can be administered or withdrawn as needed, unlike the manipulation of protein expression. In addition, NCX inhibition may also be anti-arrhythmic by preventing triggered activity due to delayed and early afterdepolarisations where NCX is thought to play an important role in their initiation [53]. Furthermore, under normal condition, the NCX inhibition has negligible proarrhythmic effects since it did not or marginally influence the ECG, the shape of the action potential and the kinetics of the Ca^2+^ transient [6,54,55].

The S1-S2 protocol has revealed that the ORM-10962 extends post-repolarization refractoriness (PRR). Computer simulations suggest the reduction in L-type calcium current following ORM-10962 exposure as a potential contributor, particularly in the setting when I_Na_ is reduced. An alternative explanation lies in the calcium-based inactivation of fast sodium current [56]: it is to be expected that following NCX blockade, sub-sarcolemmal calcium concentration would rise due to reduced calcium efflux, inhibiting fast sodium current and trivially extending PRR. Future quantitative studies may elucidate the relative contribution of these two mechanisms. Localized prolongation of PRR is known to be arrhythmogenic in the setting of acute ischemia; however, it was suggested that if PRR extension is global and without a major elevation of resting potential, it is antiarrhythmic instead, protecting the heart from re-entry [57]. PRR extension could therefore be an additional beneficial anti-arrhythmic effect of ORM-10962.

One interesting aspect of the simulation part of this study is the fact that an explicit reduction in I_CaL_ was required to manifest the experimentally observed reduction in I_CaL_ following ORM-10962 treatment. The indirect I_CaL_ reduction observed in experiments appears to stem from calcium-dependent inactivation of I_CaL_, given that the effect is abolished by the fast buffer BAPTA, or by depletion of SR [7]. At the same time, the ToR-ORd showed only a negligible reduction in peak I_CaL_ in response to pure NCX block, similarly to other models we tried this treatment on (not shown) [27,58,59]. As all these models do represent experimental data on calcium-dependent inactivation, the discrepancy between experimental results and simulations suggests a gap in our understanding of calcium-dependent inactivation of I_CaL_.

## 5 Study limitations

Our study has the following limitations:

(i) The action potential measurements were carried out on tissue while CaTs were recorded from isolated cells. This may indicate that the magnitude and kinetics of action potential alternans could not be directly related to CaT alternans. An alternative approach would be to use dual calcium-voltage imaging, but this approach would have a different set of limitations arising from the use of mechanical uncouplers such as blebbistatin [60].
(ii) The action potential measurements were performed on intact ventricular tissue to avoid shortcomings arising from repolarization attenuation caused by enzymatic dissociation. Due to the stable repolarization, the alternans could be evoked with 100% probability and small APD changes could be detected with high fidelity. At the same time, it also means that our action potential measurements represent only the endocardial tissue. Other cell layers (such as epicardial cells) may have different characteristics during alternans. Furthermore, the range of basic cycle lengths for the microelectrode measurements in ventricular tissue was determined based on pilot data suggesting that alternans will not be present at bcl of 250 ms; however, in data collected for the study, alternans was also present at bcl of 250 ms. It is consequently impossible to precisely determine the effect of ORM-10962 on alternans onset (the slowest frequency at which alternans arises). Finally, we could not pace the single cells used for calcium measurements as rapidly as the faster pacing rates in the intact preparations, given that a 2:1 block manifested; this is most likely a consequence of the cell isolation, which is known to prolong APD and thus refractoriness primarily via the reduced density of the potassium channels.
(iii) The ToR-ORd computer model does not spontaneously reduce I_CaL_ in response to NCX blockade, (similarly to [27,58,59]), and the reduction thus had to be added explicitly. While this suggests interesting avenues for further research as discussed above, it is clearly a limitation of existing models. In addition, further research is needed to create models which could manifest all three types of alternans observed in this study ('++', '+−', '+0') dependent on their parametrization, allowing further investigation into the link between CaT and APD alternans.

## 6 Conclusions

Our experimental work and computational mechanistic modelling and simulation indicate that the NCX blocker ORM-10962 can attenuate alternans in both APD and calcium transient amplitude. This is driven primarily by shifting the intracellular calcium balance to increased sarcoplasmic reticulum loading, reducing SR release refractoriness, thus attenuating alternans.

## Funding

This work was supported by grants from the 10.13039/501100011019National Research Development and Innovation Office (NKFIH PD-125402 (for NN), FK-129117 (for NN), GINOP-2.3.2-15-2016-00006 and GINOP-2.3.2-15-2016-00012), the LIVE LONGER EFOP-3.6.2-16-2017-00006 project, the János Bolyai Research Scholarship of the 10.13039/501100003825Hungarian Academy of Sciences (for NN), the EFOP 3.6.3 VEKOP-16-2017-00009 (for NT), the 10.13039/501100003825Hungarian Academy of Sciences, the 10.13039/501100000274British Heart Foundation (FS/15/8/3115 for NH and CH/2000004/12801 for DE), 10.13039/100010269Wellcome Trust (100246/Z/12/Z and 214290/Z/18/Z for BR), and by the Orion Pharma (ORM-10962).

## Contributions

Study conception: JT; Study design: JT, NN; Experimental data collection: JS, NT, RV, BH; Simulations: JT; Data analysis and visualization: JT, NN; Writing initial manuscript: JT; Critical revisions: All authors; Supervision: NN, BR, AV, NH; Funding acquisition: NN, BR, AV, NH.

## Declaration of competing interest

J. Levijoki is employed by Orion Pharma and has been involved in the development of ORM-10962. Other authors have nothing to declare

## Appendix A Supplementary data

Supplementary material Image 1
