# Supplementary material for: Blockade of sodium‑calcium exchanger via ORM-10962 attenuates cardiac alternans
Source: J Mol Cell Cardiol. 2021 Apr;153:111–22. doi: 10.1016/j.yjmcc.2020.12.015 (PMC8035081; doi:10.1016/j.yjmcc.2020.12.015)
Supplement: Supplementary file 1 — Supplementary material [file mmc1.pdf]

1 Appendices

1.1 Appendix A

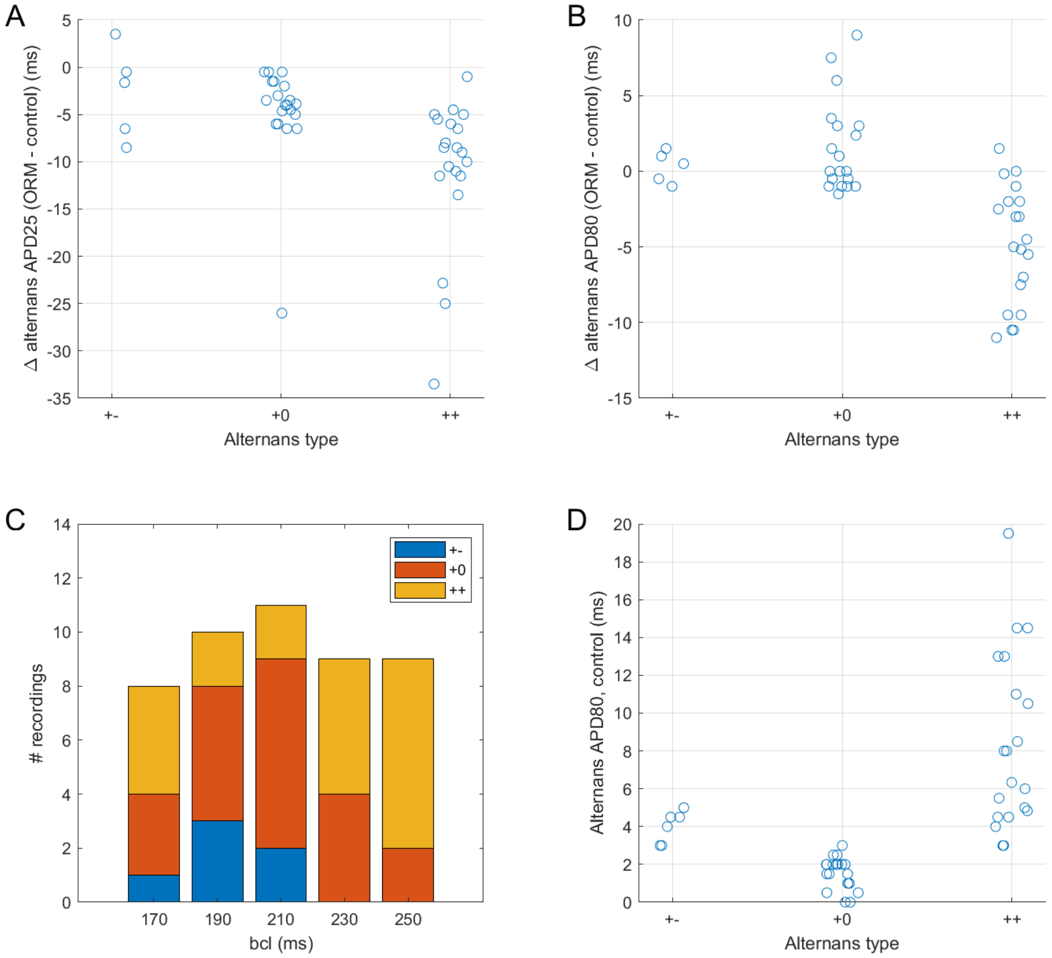

Supplementary Figure 1: Analysis of ORM-10962 effect by alternans type. A) APD25 alternans effect of ORM-10962 by alternans type (<0 corresponds to alternans attenuation, >0 to alternans promotion). **B)** APD80 alternans effect of ORM-10962 class. **C)** Number of recordings of each alternans type for the basic cycle lengths tested. **D)** APD80 alternans amplitude in control condition, separated by alternans type. We note that for A,B,D), statistical analysis was not carried out, given that data from 13 preparations and 5 basic cycle lengths were pooled, i.e., the plotted values are not all fully mutually independent.

1.2 Appendix B

We carried out additional exploration of the cases when alternans was potentiated by simulated ORM-10962 treatments (**Figure 4E**), hypothesizing that a part of the cases of potentiation could be associated with the mechanism of full sarcoplasmic reticulum (SR) depletion described previously (Tomek et al. 2018). In such a case, a control model may not manifest alternans at a given basic cycle length, because it fully depletes JSR at this frequency, but when its SR release is reduced with a treatment, it stops depleting and it allows alternans following from SR refractoriness to manifest. This would be expected to be particularly relevant for the tested conditions with lower I_{CaL} availability, given that the SR release will be attenuated in these the most. Indeed, for the five conditions shown in **Figure 4E**, 58/58, 21/21, 6/9, 4/23, and 2/3 cells showing alternans potentiation manifested full JSR depletion in control condition at the basic cycle lengths where the treated model

showed alternans, but control has not. Given that the physiological relevance of alternans attenuation via full SR depletion is debatable, this raises the possibility that even the small portion of cases where alternans was promoted by simulated ORM-10962 is potentially particular to the used type of computer model and may not occur in living cells.

1.3 Appendix C

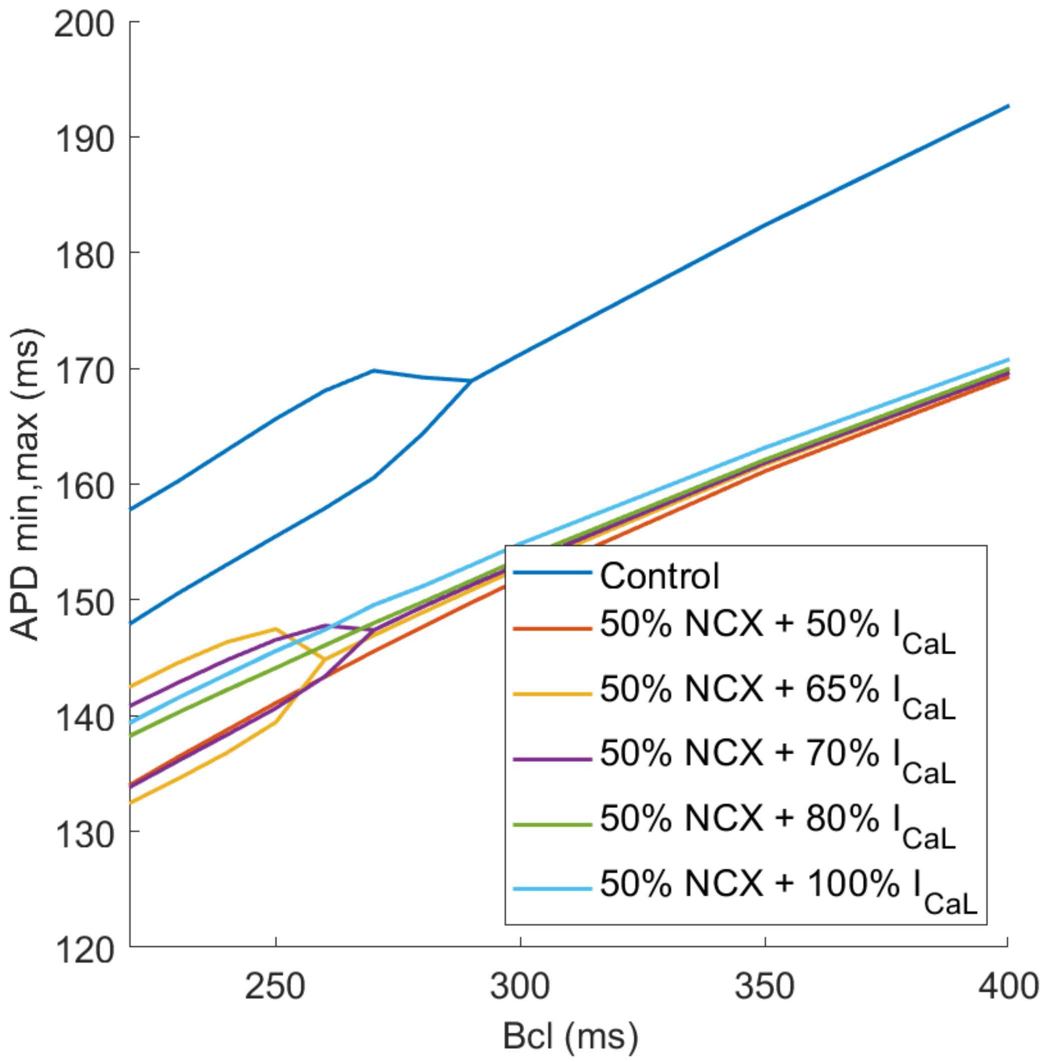

Supplementary Figure 2: Alternans at distinct basic cycle lengths for a range of combinations of reduced NCX and I_{CaL} availability. The bifurcations indicate presence of alternans, where the minimum and maximum of APD80 over two consecutive beats differs.

1.4 Appendix D

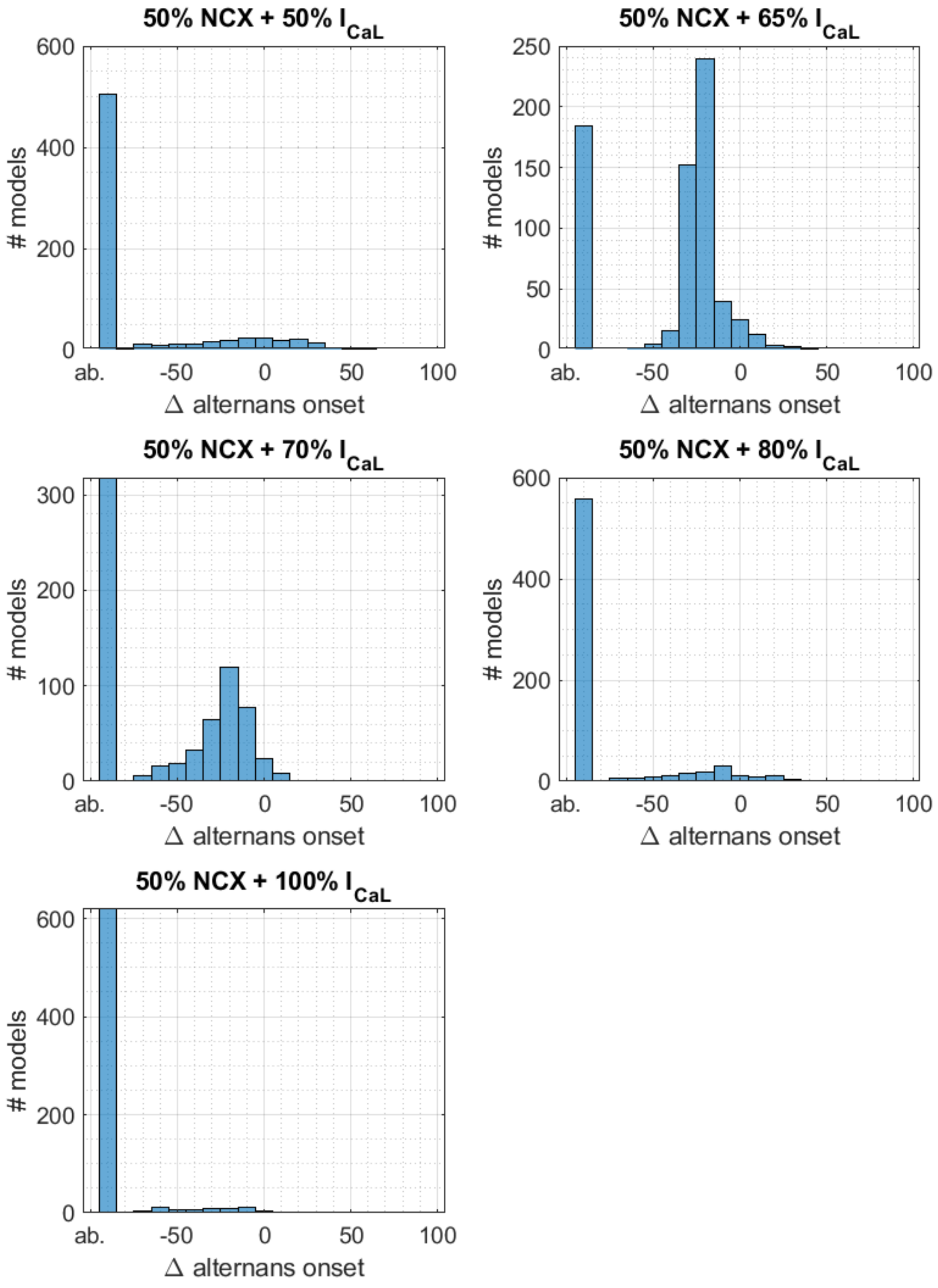

Supplementary Figure 3: Magnitude of shift in alternans onset using a population of models. For each combination of NCX and I_{CaL} availability (each in a separate histogram) and for each model out of 684, Δ alternans onset is given as the difference between alternans onset in treated cell and the control cell (alternans onset is the longest basic cycle length which manifests alternans in ms). Negative values correspond to alternans attenuation, as alternans is shifted to shorter basic cycle lengths. In many cases, alternans was completely abolished by the treatment – these cases are listed in the column 'ab.' in each panel.

1.5 Appendix E

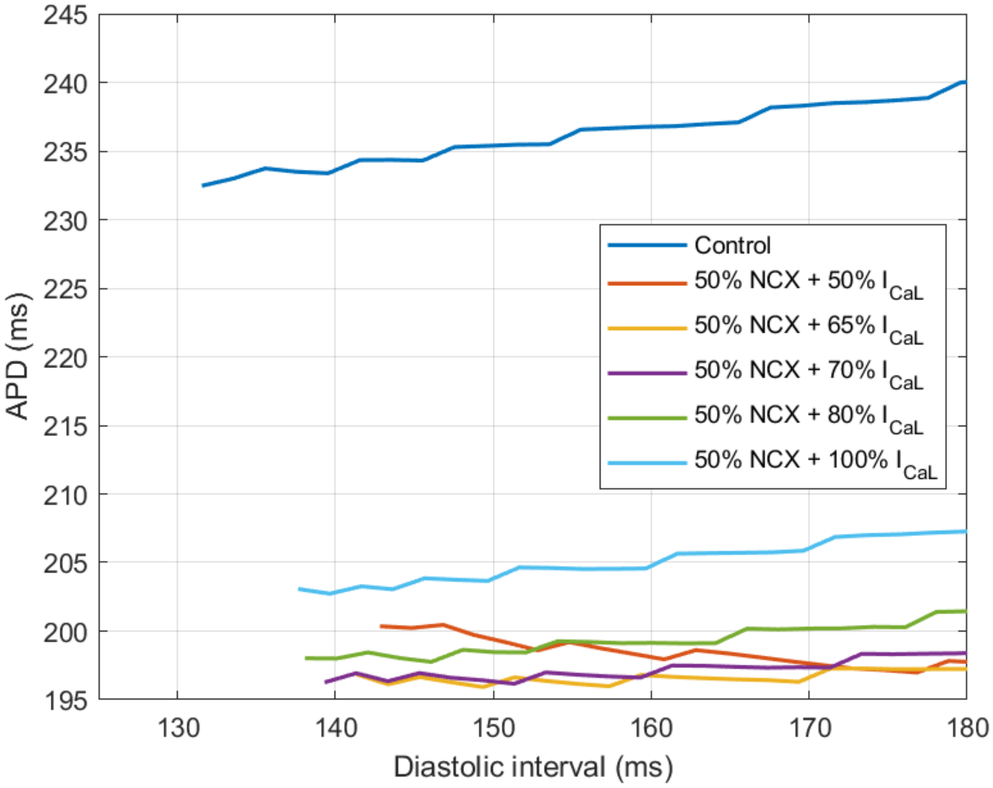

Supplementary Figure 4: Fibre simulations of S1-S2 protocol in control condition and five combinations of I_{CaL} and NCX availability. The leftmost point on each curve determines the PRR in the given condition.

1.6 Appendix F

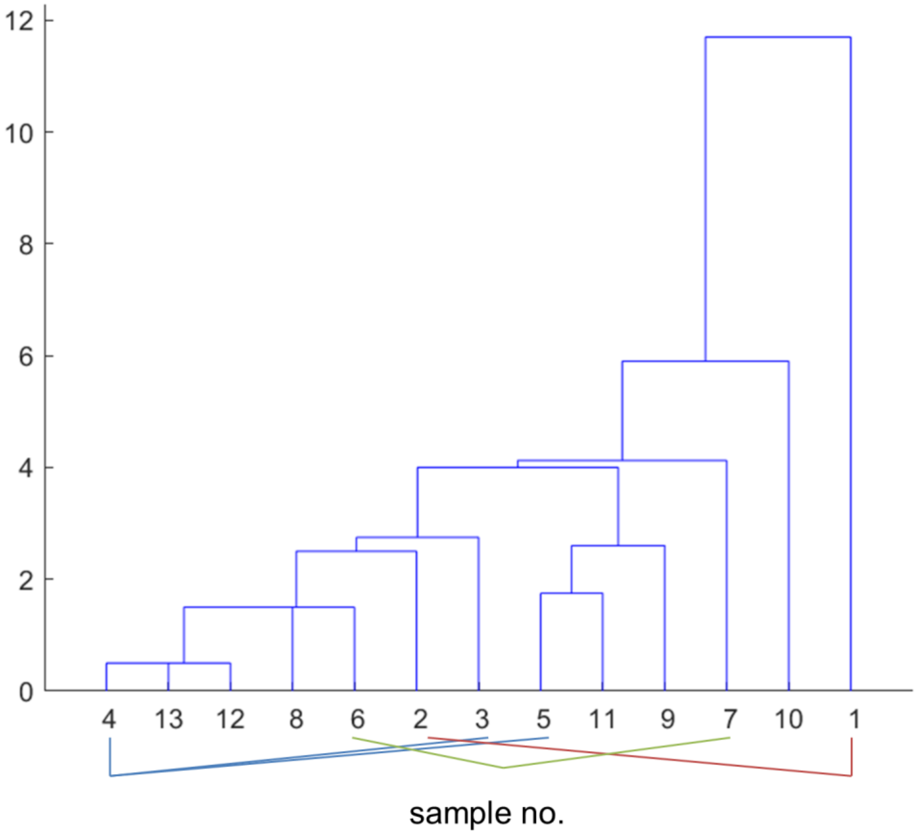

Supplementary Figure 5. Clustering of intact cardiac preparations used in Figure 1B,C. We recorded n=13 intact preparations from n=9 canine hearts. This could in principle lead to pseudoreplication if the multiple preparations from a single heart behaved near-identically, skewing the data towards artificially overrepresented behavior. To determine whether this is a problem, we performed hierarchical clustering based on a distance matrix between the 13 preparations. The distance between two preparations was defined as average difference between APD25 alternans in control condition (averaging was used over different basic cycle lengths for which data were available). I.e., if two preparations behave similarly, manifesting similar degree of alternans, they will have small distance and will cluster together. In the presented figure, samples from a single heart are linked via blue, green, and red lines, and in no case are multiple preparations from a single heart clustered together, demonstrating they are not mere pseudoreplicates.

1.7 Appendix G

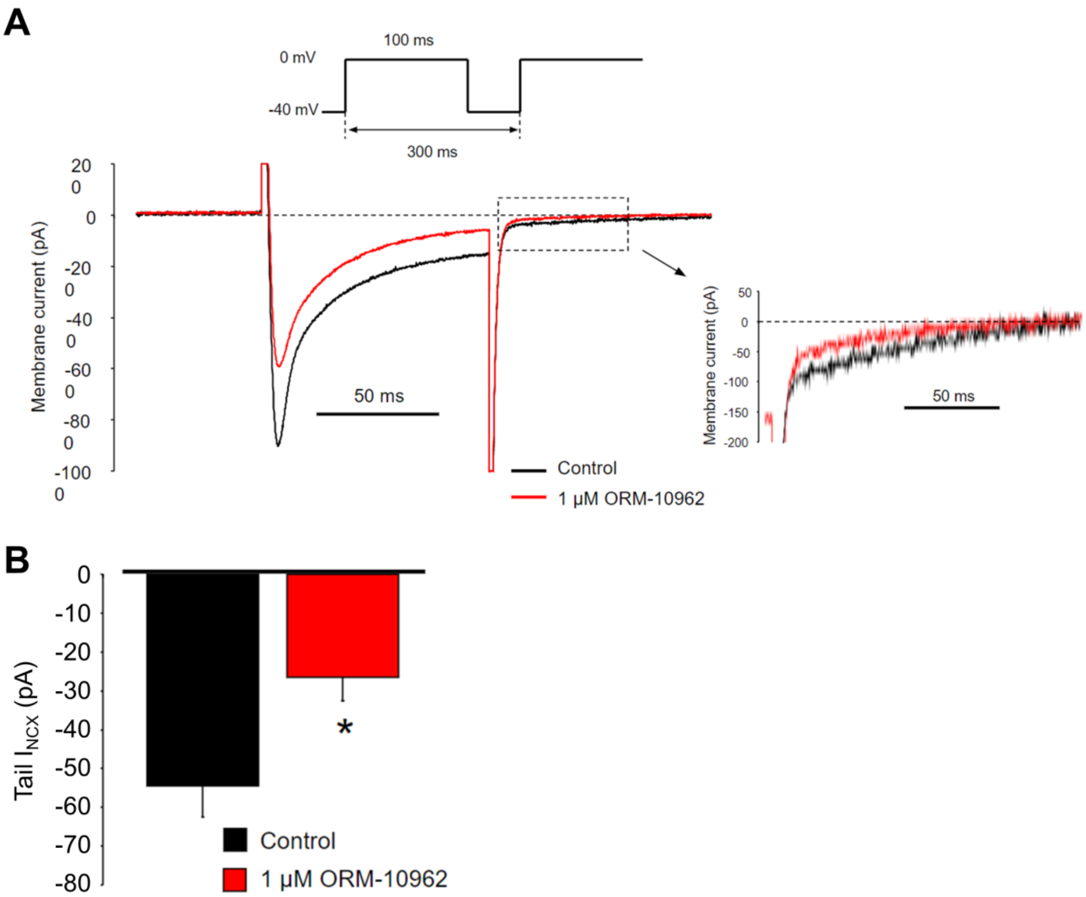

Supplementary Figure 6 Effect of ORM-10962 on NCX tail current. **A)** NCX tail in the absence (black curve) and in the presence of 1 µM ORM-10962 (red curve). The upper panel shows the applied voltage protocol having a cycle length of 300 ms. The inset illustrates the effect ORM-10962 on the NCX tail current. **B)** Effect of ORM-10962 on the NCX tail current, showing 50.7 (±5.3) % availability, consistent with [7] (n=11, p=7.25E-5). Methodology: The isolated cells were placed in a low volume imaging chamber (RC47FSLP, Warner Instruments, USA), and the cells were then continuously perfused with normal Tyrode solution at 37°C (1 mL/min). After establishing the whole-cell configuration, the external solution was switch to solution containing (in mM) 144mM NaCl, 0.4mM NaH2PO4, 4mM KCl, 0.53mM MgSO4, 1.8mM CaCl2, 5.5mM Glucose, 5mM HEPES, 3mM 4-Aminopyridine (pH 7.4 adjusted with NaOH). The pipette solution contained (in mM): 125mM CsCl, 20mM TEA-Cl, 5mM MgATP, 10mM HEPES, 10mM NaCl (pH 7.2 adjusted with CsOH). Data acquisition and analysis were performed using Axon Digidata 1550B System (Molecular Devices, Sunnyvale, CA, USA). ICaL combined with INCX was measured by rectangular voltage pulses having cycle length of 300 ms. The membrane was depolarized from a holding potential of -40 mV (to inactivate the I_{Na}) to 0 mV to initiate and inactivate I_{CaL} for 100 ms, and then was hyperpolarized to -40 mV to activate NCX tail current.
